# Balancing nitrate acquisition strategies in symbiotic legumes

**DOI:** 10.1007/s00425-023-04175-3

**Published:** 2023-06-09

**Authors:** Zainab Rahmat, Muhammad N. Sohail, Francine Perrine-Walker, Brent N. Kaiser

**Affiliations:** 1 Sydney Institute of Agriculture, The Faculty of Science, University of Sydney, 380 Werombi Road, Brownlow Hill, NSW 2570 Australia; 2 School of Natural Sciences, University of Tasmania, Private Bag 55, Hobart, TAS 7001 Australia

**Keywords:** Nitrate peptide family (NFP), Nitrogen fixation, Nodulation, Transporter

## Abstract

**Main conclusion:**

Legumes manage both symbiotic (indirect) and non-symbiotic (direct) nitrogen acquisition pathways. Understanding and optimising the direct pathway for nitrate uptake will support greater legume growth and seed yields.

**Abstract:**

Legumes have multiple pathways to acquire reduced nitrogen to grow and set seed. Apart from the symbiotic N_2_-fixation pathway involving soil-borne rhizobia bacteria, the acquisition of nitrate and ammonia from the soil can also be an important secondary nitrogen source to meet plant *N* demand. The balance in *N* delivery between symbiotic *N* (indirect) and inorganic *N* uptake (direct) remains less clear over the growing cycle and with the type of legume under cultivation. In fertile, pH balanced agricultural soils, NO_3_^−^ is often the predominant form of reduced *N* available to crop plants and will be a major contributor to whole plant *N* supply if provided at sufficient levels. The transport processes for NO_3_^−^ uptake into legume root cells and its transport between root and shoot tissues involves both high and low-affinity transport systems called HATS and LATS, respectively. These proteins are regulated by external NO_3_^−^ availability and by the N status of the cell. Other proteins also play a role in NO_3_^−^ transport, including the voltage dependent chloride/nitrate channel family (CLC) and the S-type anion channels of the SLAC/SLAH family. CLC’s are linked to NO_3_^−^ transport across the tonoplast of vacuoles and the SLAC/SLAH’s with NO_3_^−^ efflux across the plasma membrane and out of the cell. An important step in managing the *N* requirements of a plant are the mechanisms involved in root *N* uptake and the subsequent cellular distribution within the plant. In this review, we will present the current knowledge of these proteins and what is understood on how they function in key model legumes (*Lotus japonicus, Medicago truncatula* and *Glycine* sp.). The review will examine their regulation and role in *N* signalling, discuss how post-translational modification affects NO_3_^−^ transport in roots and aerial tissues and its translocation to vegetative tissues and storage/remobilization in reproductive tissues. Lastly, we will present how NO_3_^−^influences the autoregulation of nodulation and nitrogen fixation and its role in mitigating salt and other abiotic stresses.

## Introduction

**Fig. 1:**
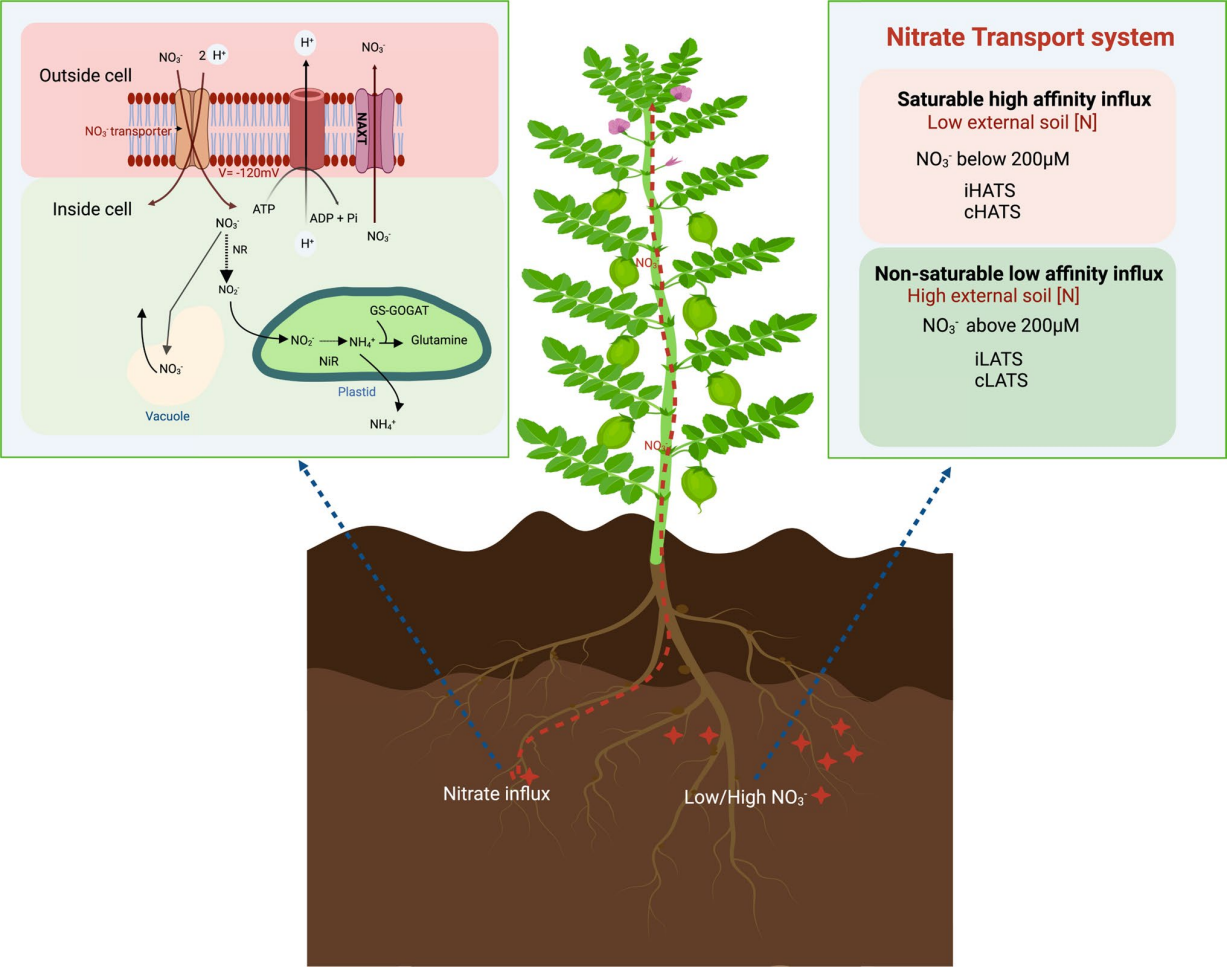
Fate of nitrate from soil to plant For NO_3_^−^ entry from soil into root cells, a P-type H-ATPase in the plasma membrane pumps protons (H^+^) out from the root cell generating an electrical gradient which helps cotransport NO_3_^−^ into the cell alongside two H^+^ ions. Inside the cell, NO_3_^−^ is transported across the tonoplast and stored in the vacuole or effluxed back to the cell apoplast with the transport across the PM though NAXT proteins. In the cytosol, nitrate reductase activity reduces NO_3_^−^ to NO_2_^−^ which then enters plastids and is reduced to NH_3_ by nitrite reductase. NH_3_ is then converted to glutamate for glutamine production. This influx of NO_3_^−^ follows biphasic pattern mostly comprising HATS (High affinity transport system) at lower soil concentration and LATS (Low affinity transport system) at higher external soil concentration

Nitrate (NO_3_^−^) transport by plants is managed through a range of concentration dependent transport proteins (Crawford and Glass 1998; Glass et al. 2002). Based on substrate affinities, NO_3_^−^ transport proteins are mainly categorized into two broad groups: (1) High Affinity Transport Systems (HATS) that are energetically dependent and active at low concentrations and (2) the more passive Low Affinity Transport Systems (LATS) driven by large chemical gradients. The majority of HATS genes and encoded proteins are activated when soil NO_3_^−^ concentrations are low (generally from 1 to 0.5 mM) (Glass et al. 2002). In contrast, the LATS proteins are generally constitutively active when NO_3_^−^ concentrations are high (exceeding 0.5 mM) (Crawford and Glass 1998; Glass et al. 2002). In addition to influx, plants also need NO_3_^−^ efflux mechanisms to help maintain internal *N* levels depending on the external environmental conditions including net *N* availability or supply. (Aslam et al. 1996; Crawford and Glass 1998; Miller et al. 2007). Under optimum environmental conditions, the rate of influx into root cells is always higher than the rate of efflux to meet the significant demands of *N* for plant growth (Kronzucker et al. 1999). When entering root epidermal and cortical cells, soil NO_3_^−^ must first traverse the plasma membrane (PM) to be utilised. The transport process is mostly an energy dependent (active) process (Aslam et al. 1996; Siddiqi et al. 1990) involving a 2H^+^/1NO_3_^−^ symport mechanism (McClure et al. 1990; Meharg and Blatt 1995; Miller et al. 2007). Once transferred across the PM, NO_3_^−^ can undergo either reduction, vacuolar storage, translocation to aerial tissues via the xylem or efflux back into the root apoplast or soil solution (Crawford and Glass 1998; Dechorgnat et al. 2011). Nitrate efflux systems have been identified in plant roots generally in response to ATP-dependent H^+^-transport activity on the PM and the resulting acidification of the apoplast. The anion channel (SLAH3) has been linked to a NO_3_^−^ efflux activity in response to ammonium (NH_4_^+^) toxicities and the subsequent acidification of the apoplast, while the NO_3_^−^/peptide transporter NAXT allows for passive NO_3_^−^ efflux across the PM in response to increased acidities around the roots (Segonzac et al. 2007; Zheng et al. 2015) (Fig. 1).

The diversity of physiological responses to NO_3_^−^ indicates that both the HATS and LATS activities are represented by different classes of transport proteins and plant-dependent functions that are required at different stages of plant development and in response to supply or concentration of NO_3_^−^ or other ions to roots and within cells (Amarasinghe et al. 1998; Crawford and Glass 1998; Glass et al. 1992; Grouzis et al. 1997; Hole et al. 1990; Martinez et al. 2015) (Fig. 1). Coordination of these activities is important to maintain N homeostasis for plant growth and development. An interesting relationship occurs when other forms of viable reduced *N* is made available to legumes (i.e., through symbiotic associations) where homeostatic balance in *N* supply (direct supply) is offset against the *N*-dependent regulatory controls of the biological N_2_-fixing or acquisition symbiosis.

Legumes can survive in *N* depleted soils via symbiosis with compatible soil rhizobia. The symbiosis results in the development of root nodules that house N_2_-fixing bacteroids within a cellular environment conducive to the fixation of atmospheric N_2_ to NH_3_ (Herridge et al. 2008; Udvardi and Poole 2013; Downie 2014). Alternatively, N_2_-fixing roots can accumulate inorganic N (NO_3_^−^ and NH_4_^+^) to support their *N* needs independent of the existing symbiosis. Furthermore, high levels of NO_3_^−^ and or ammonium (NH_4_^+^) in the soil actively inhibits symbiotic N_2_-fixation fixation (SNF), which could be due to the activity of NO_3_^−^ and NH_4_^+^ (AMT) transporters in legume roots and nodules (Ma and Chen, 2021). This dichotomy in *N* acquisition strategy provides flexibility to legumes to secure N to meet plant demand and to adjust to fluctuations in the availability of N in the soil. However, this also creates difficulties in managing the symbiotic partnership, which is negatively impacted by the concentration of reduced *N* in the soil (Concha and Doerner 2020; Nishida et al. 2018; Glian'ko et al. 2009). This conundrum forces legumes to find a genetic balance between the effective use of plant resources to acquire atmospheric N_2_ or simply rely on soil *N* without the cost of supporting a symbiosis. This becomes important in young plants prior to the establishment of an effective N_2_-fixing symbiosis, where high concentrations of soil *N* promotes growth while disrupting nodulation and potentially long-term N_2_-fxation capacity of the plant (Motte et al. 2019).

Interestingly, work by Guinet et al. (2018) highlighted the differential responses among ten legume crops to *N* fertilisation and the variation in symbiotic nitrogen fixation (SNF) inhibition. There were differences in inorganic *N* uptake among the different legume species in field experiments, which was positively correlated to rapid lateral root expansion and soil colonisation (Guinet et al. 2018). Several studies involving transcriptomic analysis have shown induction of transporter genes in nodules, particularly HATS and LATS genes (Pellizzaro et al. 2017, 2014; Valkov et al. 2017, 2020; Vittozzi et al. 2021; Wang et al. 2020; You et al. 2020) although roles of these proteins in SNF and nodulation has been reported for only a few of them. Thus, understanding how NO_3_^−^ uptake occurs and how it controls plant growth is important for optimisation of legume N inputs across the development cycle.

The rate of NO_3_^−^ uptake depends on the concentration of NO_3_^−^ in the soil, the stage of plant development and the extent of plant *N* demand (Imsande and Touraine 1994). In this review, we will provide an overview of the NO_3_^−^ transport families (NPF, NRT2, NRT3, CLC, SLAC1/SLAH3) and their involvement in NO_3_^−^ transport in plants and will unravel those activities previously characterised in the model legumes, *Medicago truncatula* (*Medicago*), *Lotus japonicus* (*Lotus*) and *Glycine max* (soybean). The review will then explore signalling activities of these transporters for root development and nodulation, their post-translational regulation and role of these transporters in legume nodules and their influence on N_2_-fixation, and nodule *N* homeostasis. Tissue NO_3_^−^ transport, storage and redistribution will be investigated and their role in the mitigation of different abiotic stresses. We hope this review will highlight where the research gaps exist in this field and where future research is required to better understand this alternative N uptake pathway operating in all legumes.

### Molecular basis for NO_3_^−^ transport in legumes

**Fig. 2:**
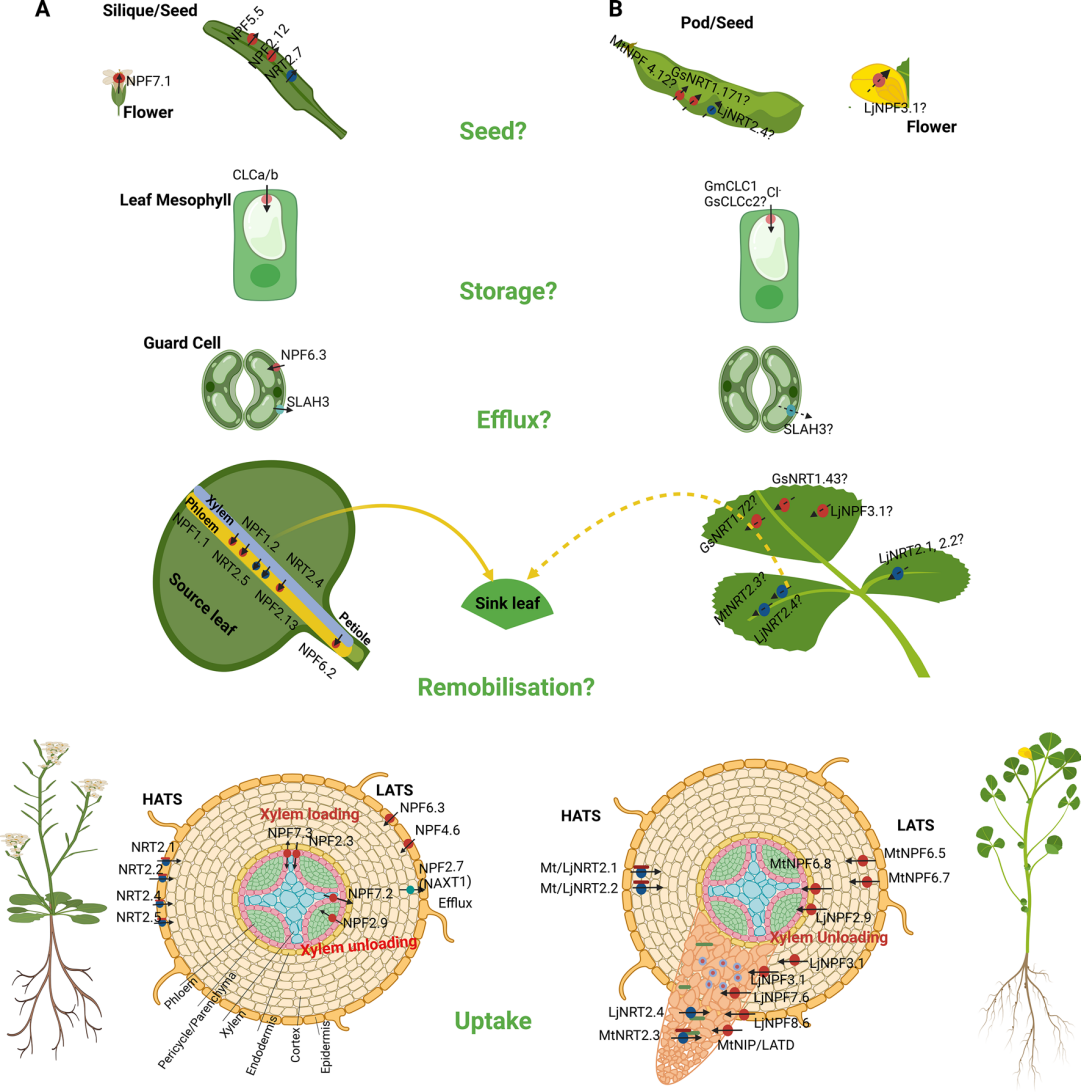
Nitrate transporters in *Arabidopsis* and legume. **A** Classes of nitrate transporters: NO_3_^−^ transporter 1 (NRT1/NPF), NRT2, chloride channel (CLC) a/b, and slow anion channel-associated 1 homolog 3 (SLAH3) to different steps of NO_3_^−^ uptake and allocation. Functions depicted include: (i) Root NO_3_^−^ uptake (NPF6.3 (CHL15/NRT1.1), NPF4.6, NRT2.1, NRT2.2, NRT2.5 and NRT2.4); (ii) NO_3_^−^ efflux (NPF 2.7 (NAXT1)). Xylem loading (NPF2.3, NPF7.3) and unloading (NPF7.2, NPF 2.9); (iii) phloem loading NPF2.3, NPF 1.1, NPF 1.2, NPF 6.2 (petiole) NRT2.4, NRT2.5, play a role in NO_3_^−^ transfer from xylem to phloem in old leaves which is then remobilised to sink leaves/ where needed. Whereas NRT2.7, NPF2.12 and NPF 5.5 play a role in seed storage of NO_3_^−^. In flowers NPF7.1 transports nitrate to pollen and the anther. CLCa/b helps in excess NO_3_^−^ storage into vacuoles. Efflux of NO_3_^−^ is regulated by SLAH3. Most NRT2 proteins interact with NAR2.1 to be functional; **B** Nitrate transporters and their role in root and nodule uptake with identified research gaps in NO_3_^−^ remobilisation, storage and efflux in legumes. Root NO_3_^−^ uptake (MtNPF6.8, MtNPF6.5, MtNPF6.7, LjNRT2.1, LjNRT2.2, MtNRT2.1, MtNRT2.2), LjNPF2.9 in xylem unloading (ii) Nodule NO_3_^−^ uptake (LjNPF8.6, MtNPF7.6, LjNPF3.1, LjNRT2.4, MtNRT2.3, MtNIP/LATD) (iii) Putative role of GsNRT1.43, GsNRT1.72, LjNPF3.1, MtNRT2.3, LjNRT2.1, LjNRT2.2, LjNRT2.4 in NO_3_^−^ remobilisation in leaves, GsNRT1.71,LjNRT2.4, MtNPF4.12 in seeds/pods and LjNF3.1 in flowers. Gm/GsCLC2 members transport Cl^−^ and controls also NO_3_^−^/ Cl^−^ hence might involve in NO_3_^−^ storage. SLAH3 (efflux) yet to be identified in legumes. (Dotted arrows indicate putative roles and ‘?’ indicate research gaps in legumes relevant to Arabidopsis)

Model plant and crop genome sequencing studies indicate that NO_3_^−^ transporters are divided into four general classifications. The first is the large Nitrate Transporter 1/Peptide Transporter (NRT1/PTR/NPF) family (Léran et al. 2014) often linked to LATS and/or dual-affinity transport activities. Many of the NPF group show an ability to transport multiple substrates and potential functions within the cell (Corratgé-Faillie and Lacombe 2017). The second is the high-affinity (HATS) Nitrate Transporter 2 family (NRT2/NNP) (Tsay et al. 2007; Dechorgnat et al. 2011). The other two families are the Chloride Channel (CLC) (Barbier-Brygoo et al. 2011) and the Slow Anion Channel-Associated Homologues (SLAC/SLAH) (Negi et al. 2008) (Fig. 2). A small number of these transporters have emerged as primary mechanisms responsible for NO_3_^−^ transport across a range of plant cellular membranes and tissues, while some are involved specifically in NO_3_^−^ transport involving both HATS and LATS activities that may be linked to NO_3_^−^ signalling (Nacry et al. 2013). With each family only a few so far have been studied in crop legumes.

### The low affinity No_3_^−^ Peptide Family (NPF)

In *Arabidopsis*, 53 genes have been identified that belong to the NPF group (Léran et al. 2014). These genes are further divided into eight subfamilies *NPF1-8* where most members are assumed to act as LATS proteins with a few notable exceptions (Liu et al. 1999; Morère-Le Paven et al. 2011; Wang et al. 2018). The common predicted structure of NPF proteins, includes 12 transmembrane domains connected with short peptide loops and a large hydrophilic loop present between transmembrane domains 6 and 7 (Tsay et al. 2007). The first identified member of this family is *NRT1.1/CHL1* (Tsay et al. 1993), which was re-classified as *AtNPF6.3*, based on the unified nomenclature used for all NRT1/PTR proteins (Léran et al. 2014). Of the few members of the NPF family which have been functionally characterised, NPF6.3-like proteins appear to transport a range of molecules, including NO_3_^−^, chloride, auxin, abscisic acid and glucosinolates (Kanno et al. 2012; Léran et al. 2014; Wen et al. 2017; Corratgé-Faillie and Lacombe 2017). Similar orthologs of AtNPF6.3 are also found and characterised as NO_3_^−^ transporters in other plant species such as rice (OsNPF6.5b), maize (ZmNPF6.4*,* ZmNPF6.6) and tomato (LeNrtl-l and LeNrtl-2) (Lauter et al. 1996; Wang et al. 2018; Wen et al. 2017; Fan et al. 2016). In *Arabidopsis,* 18 of the 53 putative NPF members are characterized as NO_3_^−^ and/or dipeptide transporters (Hsu and Tsay 2013; Wang et al. 2012; Noguero and Lacombe 2016). Other members of the non-NPF6.3 group, include NPF4.6 which acts as pure constitutive LATS for NO_3_^−^ influx (Huang et al. 1999), while NPF2.7 is involved in the efflux of NO_3_^−^ from mature root cortical cells (Huang et al. 1999; Segonzac et al. 2007; Kanno et al. 2012). The rest of the characterised NPF members (NPF7.3, NPF 7.2, NPF 2.9, NPF2.3, NPF 1.1, NPF 1.2 and NPF 6.2 reviewed by (O'Brien et al. 2016) are mainly involved in NO_3_^−^ transport within the plant (Chiu et al. 2004; Fan et al. 2009; Hsu and Tsay 2013; Taochy et al. 2015) (Fig. 2).

Genomic analysis of NPF sequences from 42 plant genomes identified a total of 43, 44, 92 and 114 NPFs from chickpea, *Lotus* and *Medicago* and soybean, respectively (Longo et al. 2018). The majority of the NPF sequences contained two conserved ExxER/K motifs that are required for proton and NO_3_^−^ transport, while for other NPF’s the motif was missing suggesting an alternative transport functionality as passive NO_3_^−^ or Cl^−^ efflux proteins (Longo et al. 2018). It will be interesting to investigate how biochemically and structurally different the NPFs are in legumes relative to other plant species and to define the individual substrate selectivity and affinity of each identified NPF active in legume roots and nodules.

In legumes, most of our understanding about NPF activity is derived from the sequenced model legumes *Lotus* and *Medicago*. Genome wide transcriptomic analysis has identified a significant number of these NPF genes are expressed in N_2_-fixing nodules (Takanashi et al. 2012). Molecular characterisation of the NPF family in *L. japonicus* identified 37 putative *LjNPF* sequences (Criscuolo et al. 2012) and at least eight members of *NPFs* are sub-classified as nodule-induced (NI) NO_3_^−^ transporter genes (Valkov and Chiurazzi 2014). One of these, LjNPF8.6 has been reported as a NO_3_^−^ transporter with potential alignment with nodule nitrogenase activity and ROS management.

LjNPF2.9, a putative LATS is involved in downward NO_3_^−^ transport from shoots to the roots via a xylem to phloem loading mediated activity (Sol et al. 2019). The recent characterisation of the root and nodule cortical expressed *LjNPF3.1* revealed an involvement in nodule activity when grown under low NO_3_^−^ concentrations (Vittozzi et al. 2021). A loss of function of *Ljnpf3.*1 reduces shoot development possibly through a reduction of root based NO_3_^−^ supply. In *M. truncatula*, annotation of the sequenced genome identified 97 putative *MtNPF* genes that encoded proteins ranging from 388 to 647 amino acids in size (Léran et al. 2014; Tang et al. 2014). The in silico expression analysis of 44 *MtNPF’s* suggested that the transporter proteins belong to the same subfamily but do not necessarily share the same function or expression profile (Pellizzaro et al. 2017). For example, *MtNPF4.7* was specifically expressed in root nodules but another member from the same subfamily (*MtNPF4.12*) was expressed during seed development. In *Glycine soja*, expression of *GsNRT1.57*, *GsNRT1.96 (NPF 7)*, *GsNRT1.84 (NPF1)* are induced by *N* supply (You et al. 2020).

The majority of MtNFP proteins have yet to be characterised though most are thought to behave as LATS proteins. In *Lotus* both LjNFP8.6 (Valkov et al. 2017) and LjNPF2.9 (Sol et al. 2019) are involved in root NO_3_^−^ uptake as LATS proteins under high NO_3_^−^ supply. However, exceptions have been reported demonstrating both low and/or high-affinity transport characteristics as reported by Liu et al. (1999) with AtNPF6;3 (AtNRT1;1). MtNPF6.8 (MtNRT1.3) shows dual-affinity transport activities (Morère-Le Paven et al. 2011). Under both low and high NO_3_^−^ supply, MtNPF6.8 (MtNRT1;3) enhances NO_3_^−^ flux into *Xenopus laevis* oocytes. In plants, *MtNPF6.8* expression in roots is enhanced when NO_3_^−^ is absent in the growth media and repressed when present. MtNPF6.8 is also considered a transceptor by its ability to also mediate ABA transport and regulate primary root growth (Pellizzaro et al. 2014). Another example of a non-LATS NFP involves the nodulation mutant in *Medicago* (*Mtnip-1*), which shows higher nodulation sensitivity to NO_3_^−^ than WT plants. *Mtnip-1* contains a lesion in the gene *MtNIP/LATD (MtNPF1.7)*, which is an *NPF* ortholog (Bagchi et al. 2012). When expressed in oocytes, MtNPF1.7 acted as a high-affinity NO_3_^−^ transporter in a pH dependent manner, indicating a H^+^ dependent symport of NO_3_^−^ influx. A second NPF to show high-affinity NO_3_^−^ transport is MtNPF7.6 (Wang et al. 2020). NPF proteins are also involved in plant chloride uptake. Recently two orthologues of AtNPF6.3 (MtNPF6.5 and MtNPF6.7) were identified in *M. truncutala*, showing an ability to transport both NO_3_^−^ and Cl^−^ (Xiao et al. 2021) in a similar fashion to that previously observed by ZmNPF6.4 and ZmNPF6.6 (Wen et al. 2017). However, MtNPF6.5 showed Cl^−^ selectivity whereas its close homologue MtNPF6.7 preferred NO_3_^−^ over Cl^−^.

In nodules, the high-affinity NO_3_^−^ transporter *MtNPF7.6* is expressed in vascular transfer cells (Wang et al. 2020). Mutants (*Mtnpf7.6)* show defects in nodule vasculature development and a reduction in bacteroid nitrogenase activity. *MtNPF7.6* is suggested to mediate NO_3_^−^ uptake from the soil solution to deliver low concentrations to developing nodules through xylem to phloem transfer in vascular tissues. The rate and quantity of NO_3_^−^ transport into nodules influences nodule development, possibly through mechanisms involving NO influence of LB activity and oxygen delivery to the bacteroids (Kanayama et al. 1990). A similar mechanism was reported in rice which showed OsNPF2.2’s role in vasculature development of different organs in rice (Li et al. 2015). MtNPF1.7 (MtLATD/NIP), another high-affinity NO_3_^−^ transporter was also reported to have a role in nodulation (Bagchi et al. 2012; Yendrek et al. 2010) where mutant plants (*Mtlatd*) showed defective nodule development (Yendrek et al. 2010; Bright et al. 2005).

The low-affinity NO_3_^−^ transporter *LjNPF8.6* is strongly expressed in mature nodules (Valkov et al. 2017). Loss of function mutants (*Ljnpf8.6*) show a reduction in N_2_-fixation but no change to nodulation or nodule number. *LjNPF3.1* is expressed in roots and in the nodule outer cortex (Vittozzi et al. 2021). The loss of *Ljnpf3.1* shows a reduction in nodule growth and N_2_-fixation activity when supplied no or low concentrations of NO_3_^−^ (< 1 mM). Shoot growth in the mutant was compromised but could be recovered at elevated NO_3_^−^ supply (5 mM).

### The high-affinity NRT2 family

The members of the NRT2 family are responsible for high-affinity NO_3_^−^ transport (HATS) in plants. NRT2 transporters share structure similarity with NPFs having 12 transmembrane regions with a large hydrophilic loop between TM 6 and TM 7, although both families do not share sequence homology (Von Wittgenstein et al. 2014). In *Arabidopsis*, seven members have been characterized as HATS transporters (Kotur et al. 2012; Krapp et al. 2014). The four members (NRT2.1, NRT2.2, NRT2.4, and NRT2.5) are found to be involved in root NO_3_^−^ uptake under N deficient conditions (Kiba et al. 2012; Kiba and Krapp 2016; Lezhneva et al. 2014; Orsel et al. 2002). However, studies have shown that among the majority of plant species, NRT2.1 activity is the main component of HATS for root NO_3_^−^ uptake (Garnett et al. 2013; Cerezo et al. 2001; Filleur and Daniel-Vedele 1999; Li et al. 2007; Miller et al. 2007). In other crops like maize, two high-affinity nitrate transporters, ZmNRT2.1 and ZmNRT2.2 were found to respond to developmental changes in NO_3_^−^ uptake and demand (Garnett et al. 2013). NRT2.1 activity has also been linked to plant hydraulic conductance in *Arabidopsis*, (Li et al. 2016). AtNRT2.4 and AtNRT2.5 are involved in phloem uploading of NO_3_^−^ and are expressed in shoots (Kiba et al. 2012; Lezhneva et al. 2014) whereas AtNRT2.7 is found to be a major contributor of NO_3_^−^ storage in seeds (Chopin et al. 2007).

In legumes very few NRT2 transporters have been functionally characterised. Amarasinghe et al. (1998) found that the expression of the soybean high-affinity NO_3_^−^ transporter (*GmNRT2*) was higher in plants grown with NO_3_^−^ compared to *N*-deprived conditions. The expression of *GmNRT2.1* and *GmNRT2.2* has since been shown to be in the exodermis and epidermis of soybean roots, a similar expression pattern to *AtNRT2.1* and *AtNRT2.4* (Peng et al. 2019; Kiba et al. 2012; Li et al. 2007). In both *Lotus* and *Medicago*, NRT2 genes have been identified and partially characterised (Criscuolo et al. 2012; Pellizzaro et al. 2014). Analysis of the *Lotus* genome identified four putative *LjNRT2* genes named *LjNRT2.1*, *LjNRT2.2*, *CM0001.20* and *CM0161.180* (*LjNRT2.7*). Two NRT2 genes (*LjNRT2.1* and *CM0161.180* (*LjNRT2.7*)) showed strong induction while the other two genes (*LjNRT2.2* and *CM0001.20*) showed no response to NO_3_^−^ (Criscuolo et al. 2012).

Recently (Valkov et al. 2020) found that LjNRT2.4 activity is positively linked to N_2_-fixation capacity and NO_3_^−^ accumulation in *Lotus* nodules. *LjNRT2.4* is expressed in both the nodule and root vascular tissues. When grown on low NO_3_^−^ concentrations (100 μM) under both symbiotic and non-symbiotic conditions, *Ljnrt2.4* mutants displayed significant reduction in shoot biomass, NO_3_^−^ content and nitrogenase activity in nodules compared to the inoculated wild type. In *Medicago,* three *NRT2* genes (*MtNRT2.1*, *MtNRT2.2* and *MtNRT2.3*) have been identified with varied expression in roots, shoots and nodules (Pellizzaro et al. 2015). *MtNRT2.1* shows typical HATS expression profiles with an induction in response to NO_3_^−^ supply. *MtNRT2.1* expression is higher in roots than shoots and more specifically in lateral roots. In contrast, *MtNRT2.3* expression is constitutive, and *MtNRT2.2* expression barely detectable. All three genes were expressed in nodules but at low levels. *MtNRT2.3* expression is enhanced in nodules (relative to root tissues) under minus N conditions or when supplied NO_3_^−^ (Pellizzaro et al. 2015). Under limited N supply, both *MtNRT2.1/MtNAR2* (Krouk et al. 2006; Pellizzaro et al. 2015) and *LjNRT2.1* are the major expressed genes and most likely contributors to the HATS component of root NO_3_^−^ uptake in these plants (Criscuolo et al. 2012). In *Glycine soja, GsNRT2.2* and *GsNRT2.4* were found upregulated when grown without N (You et al. 2020).

### The NRT2 facilitator, NRT3

For NRT2 proteins to transport NO_3_^−^ they require physical interaction with the small partner protein, NAR2 (NRT3) (Okamoto et al. 2006). NRT3 members are thought to play a role in localisation and stabilisation of NRT2.1 at the plasma membrane and facilitating NO_3_^−^ influx (Wirth et al. 2007). It has been proposed that the functional unit is composed of a NRT2 dimer and an NAR2 dimer, forming a heterotetrameric protein complex (Kotur and Glass 2015). The functional activity of NAR2 was first identified in *Chlamydomonas reinhardtii*, where co-expression of *CrNAR2*, *CrNRT2.1* and *CrNRT2.2* enhanced NO_3_^−^ transport in *Xenopus laevis* oocytes (Zhou et al. 2000). In *Arabidopsis*, a similar functional dependence on NAR2 has been shown for most of NRT2 proteins (AtNRT2.1, AtNRT2.2, AtNRT2.4, and AtNRT 2.5) except for AtNRT2.7 (Kotur et al. 2012; Kotur and Glass 2015). In other crops like wheat (TaNRT2.1/TaNRT3.1), rice (OsNRT2.2/OsNAR2.1) and maize (ZmNRT2.1/ZmNRT3.1) these partner proteins interact with NRT2 as major components of the HATS transport activity (Taulemesse et al. 2015; Feng et al. 2011; Yan et al. 2011; Chen et al. 2016; Lupini et al. 2016). In *Medicago*, genome analysis reveals two NAR-like genes, *MtNAR2.1* and *MtNAR2.2* (Pellizzaro et al. 2015). A similar gene is also found in *L. japonicus* (*LjNAR2.1*) (Criscuolo et al. 2012). A recent study in *M. truncatula* revealed that *M*t*NRT3.1* may also act as target nitrate transporter which helps to mitigate arsenic accumulation in legume crops through an ABA/NO_3_^−^ signalling mechanism (Ye et al. 2021). This suggests for the first time other potential roles that the NRT3 family performs in plants.

### Intercellular NO_3_^−^ transport, CLC

After the entry of NO_3_^−^ into cells via NRT2 and NPF family members, NO_3_^−^ can be assimilated via nitrate reductase or be partitioned to the vacuole where concentrations can increase above 50 mM (Martinoia et al. 2000; Miller and Smith 1992). This process helps to maintain cytosolic NO_3_^−^ homeostasis and provides a mechanism for osmotic balance of the cell (Cookson et al. 2005). Transport across the tonoplast has been shown to involve a voltage dependent Cl^−^/NO_3_^−^ channel family (CLC). CLC genes have been found in various plants with seven CLC homologues identified in *Arabidopsis* (Hechenberger et al. 1996; Li et al. 2006; Lurin et al. 1996; Wang et al. 2015; Wei et al. 2013; Zhou et al. 2013; Lv et al. 2009). AtCLCa acts as a two-anion/H^+^ exchanger with higher selectivity to NO_3_^−^ over Cl^−^ that helps keep NO_3_^−^ levels normal (De Angeli et al. 2006) and it also plays an important role in the opening of stomata through adjustments in osmotic potentials in the cell (Wege et al. 2014). AtCLCb functions as an NO_3_^−^/H^+^ antiporter on the tonoplast of the vacuole (von der Fecht-Bartenbach et al. 2010).

In legumes, GmCLC1 is located on the tonoplast and is induced by water and NaCl stress in soybean leaves and roots (Li et al. 2006; Wong et al. 2013). GmCLC1 helps mitigate salt stress by limiting Cl^−^ accumulation in the shoot (Wei et al. 2016). Recently, another CLC transporter GsCLC-c2 from wild soybean (*Glycine soja*) has shown similar affinities for NO_3_^−^ and Cl^−^ anions. GcCLC-c2*’*s affinity for the Cl^−^ anion is pH independent as compared to GmCLC1, where Cl^−^ affinity was pH dependent (Wei et al. 2019; Wong et al. 2013). The overexpression of *GmCLC-c2* using hairy root transformation provided NaCl tolerance and anionic balance with increased Cl^−^ accumulation in roots which limits its transport to the shoot (Wei et al. 2019). Another recent study shows that overexpressing *GsCLC-c2* in Arabidopsis improved growth when under salt stress, indicating the importance of this protein in potential cellular osmotic balance (Liu et al. 2021). In *L. japonicus*, the expression of a putative CLC transporter (*LjCLC-B*) was downregulated alongside other NO_3_^−^ transporter related genes (*LjNRT2.1* and *LjNRT2.1*) in nodulated roots compared to roots without rhizobia inoculation (Pérez-Delgado et al. 2020). The decrease in its expression and other NO_3_^−^ transport genes may reflect a response to the N status of the experimental plants (± N_2_-fixation) or suggests a nodulation enhanced repression of gene activity. It will be important to further explore this family to examine their potential involvement in salt tolerance and/or drought and their relationship to root symbiotic N status and whether resupply of NO_3_^−^ to nodulated roots would activate their expression and activity.

### NO_3_^−^ efflux, SLAC1/SLAH3 proteins

Plant NO_3_^−^ efflux systems are linked to the S-type anion channels of the SLAC/SLAH family (Negi et al. 2008). SLAC/SLAH channels have been shown to transfer both NO_3_^−^ and Cl^−^ ions. The SLAC/SLAH family has been extensively characterised in *Arabidopsis* where 4 genes exist but have also been identified in other non-legume plants including poplar, rice and pear (Jaborsky et al. 2016; Negi et al. 2008; Sun et al. 2016; Vahisalu et al. 2008; Chen et al. 2019). In *Arabidopsis*, SLAC1 plays an important role in the regulation of stomata opening and closure in response to various environmental stimuli, including ABA and CO_2_ (Negi et al. 2008; Vahisalu et al. 2008; Chen et al. 2010). Expressed in guard cells, SLAH3 is activated by NO_3_^−^ and ABA having preference of NO_3_^−^ over other ions, which suggests this channel is responsible for stomatal closure under drought stress and also in NO_3_^−^ metabolism (Geiger et al. 2011).

In rice and pear, OsSLAC1 and PbrSLAH3 behave as NO_3_^−^ efflux channels in root cells (Sun et al. 2016; Chen et al. 2019). In poplar, SLAC homologs are involved in the night time efflux of NO_3_^−^ into the xylem (Siebrecht et al. 2003). In legumes, the expression of a putative S-type anion channel-like protein was found to be downregulated in the roots of nodulated *L. japonicus* plants (Pérez-Delgado et al. 2020). Unfortunately, the role of these transporters in legumes and the plant–microbe symbiotic relationship in nodules is unknown.

As highlighted above, only a few NO_3_^−^ transporters have been identified and characterised in legumes. Their functionality in root NO_3_^−^ uptake needs to be described further as does their role in NO_3_^−^ transport inside N_2_-fixing nodules. Two nodulins (GmN70 and LjN70) have been shown to be nitrate transporters and located on the symbiosome membrane at late stage of nodule development. Both proteins are not related to NPF or NRT2 and are able to transport both NO_3_^−^ and Cl^−1^ (Vincill et al. 2005). What role these have in anion transport across the symbiosome remains to be determined. Overall, it will be important to examine a larger profile of legume root and nodule NO_3_^−^ transporters for their role in NO_3_^−^ uptake and signalling when exposed to exogenous NO_3_^−^ in either the symbiotic or non-symbiotic state.

### NO_*3*_^*−*^ transporters and their role in N signalling activities

Apart from the physical delivery of NO_3_^−^ into and out of root cells and organelles, NO_3_^−^ has an important role in communicating *N* availability and influencing both structural changes in tissue design and the eventual expression of target genes required for acquisition, assimilation and redistribution across cells and tissues. For some time, NO_3_^−^ has been shown to act as an early signal that triggers multiple growth responses, including that of roots, leaves, flowering times, and seed dormancy (See review from Medici and Krouk (2014)). NO_3_^−^ supply can initiate primary root growth leading to the emergence and development of lateral roots (Cerezo et al. 2001; Forde and Lorenzo 2001; Vidal et al. 2010; Krouk et al. 2010; Medici and Krouk 2014; Canales et al. 2017) This primary nitrate response (PNR) is a component of the larger genetic response plant genomes mount when exposed to NO_3_^−^ from an N-deprived condition. A significant player in this response involves the NO_3_^−^ transceptor AtNPF6.3 which can both transport NO_3_^−^ but also independently orchestrate signalling pathways mediating the expression of genes including the primary NO_3_^−^ transporter NRT2.1 (Ho et al. 2009).

Many legumes can establish a symbiotic partnership with compatible soil-borne rhizobia when grown in the presence of low external *N* concentrations. NO_3_^−^ can also deregulate the rhizobia symbiosis and impact both root and nodule development in positive (low concentrations) or negative (high concentrations). The *Medicago* AtNPF6.3 ortholog, MtNPF6.8 has been reported as master nitrate signal sensor in primary root tips (Zang et al. 2022). Its central role in the primary nitrate response in *Medicago* is highlighted by the lack of a genetic or proteomic response when a *Mtnpf6.8–3* mutant is exposed to different NO_3_^−^ concentrations. Transcriptomic data revealed 7,063 genes responded to nitrate in wild-type roots, while only 297 changed in the mutant. The nitrate responsive genes NPF6.8 include *NR1, NR2, GS2, and NPF6.7* which alone are known to orchestrate root architecture (Zang et al. 2022). This is supported by previous reports where MtNPF6.8 also senses external NO_3_^−^ concentrations resulting in the regulation (inhibition) of primary root growth in a *Mtnpf6.8* mutant (Pellizzaro et al. 2014). This negative interaction may involve an ABA signal operating downstream of the primary MtNPF6.8 signal as root growth could be recovered with ABA application. In a recent study Zang et al. (2020) found that a decrease in superoxide concentrations in response to NO_3_^−^ is responsible for slow root growth and that the ROS species are working downstream of MtNPF6.8 mediated NO_3_^−^ signalling. Further analysis of ROS activity in *Mtnpf6.8–2* confirmed that NO_3_^−^ reduces ROS concentrations. MtLATD/NIP (MtNPF1.7) has also been reported to have a role in ROS homeostasis (Zhang et al. 2014). The *latd* mutant line shows increased ROS levels and reduced root growth. ABA application can rescue the *latd* phenotype through an ABA-mediated decrease in ROS. ABA independent ROS reduction in the *latd* mutant also rescued the wild-type phenotype suggesting that MtLATD/NIP (MtNPF1.7) might be involved in ABA signalling. In addition to slow root growth and development, the *latd* mutant also showed abnormal nodule development, with infection thread arrest in root hairs with a rhizobial deprived primordium. ABA application fails to rescue the *latd* phenotype, suggesting that the nitrate induced nodule regulation by MtNFP1.7 is ABA independent (Liang et al. 2007; Yendrek et al. 2010). This contrasting regulatory role of NO_3_^−^ transporters in root and nodule growth development highlights a complex role these NO_3_^−^ proteins confer to plant growth and development.

### The influence of NO_***3***_^***−***^ on nodulation

**Fig. 3:**
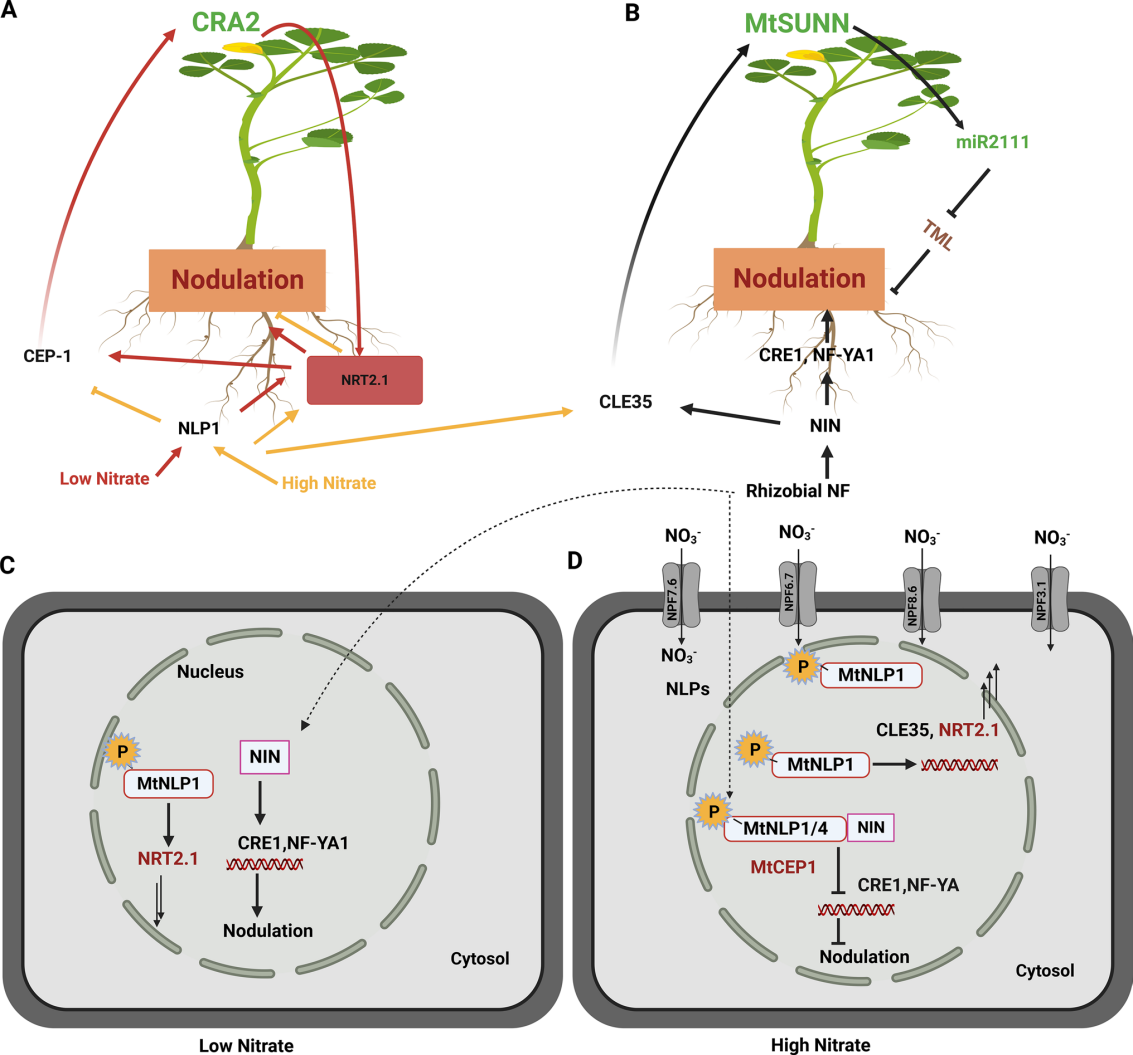
Local and systemic signalling in legumes for regulation of nodulation. Local and systemic NO_3_^−^ signalling for regulation of nodulation using NIN, NLPs under low and high *N*. **A** Low N induces *MtCEP1* expression which systemically induce *MtNRT2.1* expression through MtCRA2 in the shoot. MtNRT2.1 mediated nitrate uptake further enhances nodulation and *MtCEP1* expression (red arrows) (**B** and **C**). Under Low *N*, Nod factors induce NIN expression in the nucleus that leads to transcriptional activation of target symbiotic genes (*CRE1*, *NFY-1* and others) promoting nodulation. **C** Under low NO_3_^−^, limited nucleus localization of MtNLP1 activates low-level MtNRT2.1 expression **D**. Under high N, NLPs (MtNLP1/4) accumulates in the nucleus followed by phosphorylation, where they interact with NIN (by competing with NIN for its DNA binding sites) and supresses the expression of NIN activated symbiotic genes (*CRE1* and *NF-YA1*) disrupting nodulation. **B** and **D** MtNLP1 also activates expression of CLE like peptides such as CLE-35 in *Medicago truncutala* which are transported to the shoot and are perceived by LRR-RLK receptors, MtSUNN. This interaction leads to a reduction in levels of phloem mobile signal *miR2111* and subsequent increase levels of TML, which negatively regulates nodulation. **A** and **D** Under high N, MtNLP1 also activates MtNRT2.1 expression promoting nitrate uptake and supressing nodulation (yellow arrow)

NO_3_^−^ signalling cascades have been extensively dissected in *Arabidopsis* and studies have shown that both Ca^2+^-sensor protein kinases and NIN-like (NLP) transcription factors are involved in regulating gene expression of NO_3_^−^ transporters and assimilation genes (Liu et al 2017) (Fig. 3f). In plants, signalling pathways involve C-terminal peptides (CEPs) that act as nutritional signals produced in root vasculature cells at a N-starved site. This is followed by their movement to the shoots via the xylem. On reaching the shoot, CEPS are perceived by two leaf-specific leucine rich kinase receptors (CEPR1 and CEPR2) (Tabata et al. 2014) resulting in the induction of two shoot phloem signals, CEP downstream 1 (CEPD1) and CEPD2 (Ohkubo et al. 2017; Tabata et al. 2014). These signals travel back to the root to induce expression of the high-affinity NO_3_^−^ transporter *AtNRT2.1* (Ohkubo et al. 2017). Recently it was shown that another 2C protein phosphatase CEPH is target of CEPD, which dephosphorylates NRT2.1 at the C-terminal S501 site leading to its activation and ultimately increased NO_3_^−^ uptake and plant growth (Kaiser 2021; Ohkubo et al. 2021) In legumes, nitrate signalling also affects gains and costs associated with symbiosis and root development. Plants have developed strategies like autoregulation of nodulation (AON) to control infection and reduce the function of nodules to cope with variable *N* situations. NO_3_^−^ transporters involved in this suppression are largely unknown. AON involves the systemic long-distance signalling of peptides between root and shoot tissues. NIN-like proteins (NLPs) belonging to RWP-RK family of plant transcritiopn factors have emergered as major players of NO_3_^−^ signalling pathways by activating the expression of root derived CLE peptides*.* Nod factor induces NIN expression which transcriptionally regulates up to three CLE genes, *CLE-RS1/2/3* in roots. The encoded peptides are mobile signals that interact with respective receptors in shoots of different legume species including, the *Lotus* HYPERNODULATION ABERRANT ROOT1 (HAR1) (Nishimura et al. 2002), SUPER NUMERIC NODULES 1 (SUNN1) in *Medicago* (Schnabel et al. 2005), and NODULE AUTOREGULATION RECEPTOR KINASE1 (NARK1) in soybean (Searle et al. 2003). The signalling cascade continues with the regulation of the TOO MUCH LOVE (TML) genetic module through the expression of basipetally delivered miR2111, which can destroy TML mRNA transcripts. Rhizobial and NO_3_^−^ signals discourage miR2111 synthesis while low N enhances miR2111 synthesis (Okuma and Kawaguchi 2021). TML is a kelch repeat-containing F-box protein that can inhibit nodulation (Takahara et al. 2013) Shoot derived cytokinins also act downstream of LjHAR1 to regulate nodulation (Sasaki et al. 2014). Reduced plant growth is observed in knockout mutants of AON genes indicating the importance of this negative feedback for maintaing symbiotic balance and plant growth. In legumes, split root and grafting experiments have revealed that long-distance signalling via MtSUNN1/LjHAR1/GmNARK1 pathways also integrates plant *N* status (Jeudy et al. 2010; Okamoto and Kawaguchi 2015; Reid et al. 2011). The expression of *CLE-RS2, RS3* and *LjCLE 40* is also induced by NO_3_^−^ together with rhizobial infection (Okamoto et al. 2013) suggesting that NO_3_^−^ induced nodule inhibition shares common elements with AON (Fig. 3).

In response to NO_3_^−^, forward and reverse genetic approaches have revealed the involvement of different NLPS in nodulation regulation in *Lotus* (NRSYM1/NLP4, NRSYM2/NLP1) and *Medicago* (MtNLP1, MtNLP3 or MtNLP4) (Lin et al. 2018; Nishida et al. 2018, 2021). The uptake of NO_3_^−^ into root cells triggers the nuclear retention of NLPs, a process dependent upon the N-terminal phosphorylation of NLP Ser226. This process is conserved in many plant species including *Arabidopsis*, *Medicago* and *Lotus* (Lin et al. 2018; Liu et al. 2017; Marchive et al. 2013; Nishida et al. 2018). In *Lotus*, all five NLPs can bind to NIN-binding nucleotide sequences (NBSs) called NO_3_^−^ responsive elements (NREs) located in the promoters of genes, including *NRT2.1* and *NIR1* (Soyano et al. 2015). LjNRT2.1 plays an important role in the primary NO_3_^−^ signalling required to initiate NLP binding and activation of the AON system. Post the primary NO_3_^−^ response (NPR), accumulated NO_3_- promotes the binding of NLP1 to the promoter of *NRT2.1*, which further increases *NRT2.1* expression and consequently NO_3_- influx into the cell. The increased NO_3_^−^ concentration in the cytosol triggers a nuclear enrichment of NLP4 which can then activate the expression of NRE regulated *CLE-RS2* genes and the onset of the AON regulatory pathway (Misawa et al. 2022). In opposite fashion, rhizobial infection in roots stimulates *LjNIN* expression which can block NRT2.1 activity and disrupt the *LjCLE-RS2* signalling cascade required to activate AON. NIN expression also stimulates a number of positive regulators of nodulation (*LjNF-YA*, *LjNF-YB* and *LjEPR3*) (Misawa et al. 2022). In contrast, *GmNIC1* showed induction upon NO_3_^−^ treatment but not rhizobial infection, and acted locally to inhibit nodulation (Reid et al. 2011).

The tight regulation of NRT2.1 expression may reflect a plant strategy in which the acquisition of N switches depending on the availability of N in the soil. Similarly, in *Medicago*, NO_3_^−^ triggers the accumulation of MtNLP1 in the nucleus, which can then bind directly to the promoter of *MtCLE35* activating its expression via SUNN as a negative AON regulator of nodule number (Luo et al. 2021). In another study, MtNLP1-dependent repression of *MtNPF6.5* or enhancement of *MtNPF6.7* expression helped mediate NO_3_^−^ and Cl^−^ uptake, respectively (Xiao et al. (2021).

Another signalling pathway involving CEPs supresses lateral root formation while simultaneously positively regulating nodulation via separate mechanisms downstream of its putative CRA2 (COMPACT ROOT ARCHITECTURE 2) receptor. In *Medicago,* when *MtCEP1* was overexpressed, a decrease in lateral root number was observed, whereas knocking out *MtCEP1* and *MtCEP2* led to more lateral root numbers with less nodulation (Imin et al. 2013). Mohd-Radzman et al. (2016) revealed that MtCEP1 interacts with its putative receptor CRA2 to influence nodulation via the EIN2/SKL pathway. However biochemical proof of the physical interaction between CEP1 and CRA2 remains to be identified. Suppression of lateral root growth is done locally by CAR2, whereas its systemic long-distance signalling from shoots positively regulates nodulation. Other CEP members like MtCEP7 are also induced by rhizobia or cytokinin and its silencing by RNAi results in significantly less numbers of nodules (Laffont et al. 2020). The exogenous application of synthesised CEP peptides (MtCEP1, MtCEP2, MtCEP4, MtCEP5, MtCEP6, MtCEP8 and MtCEP12) can decrease lateral root numbers and increase nodule numbers, a result validating the previous findings of Zhu et al. (2021).

In *Medicago*, the high-affinity nitrate transporter, MtNRT2.1 has been reported recently in regulation of nodulation (Luo et al. 2023). The study proposed a MtCRA2-dependent involvement of MtNRT2.1 in both the support of nodulation and its inhibition. Under limited N supply, MtCEP1 was highly expressed, which then upregulated MtNRT2.1 expression systematically via MtCRA2 in the shoot. Furthermore, low N resulted in a reduction of the nuclear localization of MtNLP1, which activated low levels of MtNRT2.1 expression—enhancing nitrate uptake to improve plant growth and nodulation. On the other hand, ample N triggered nuclear retention of MtNLP1 resulting in CLE35 expression, which not only resulted in the negative regulation of nodulation via a SUNN dependent manner, but also activated MtNRT2.1 expression to promote nitrate uptake, and further inhibit nodulation (Luo et al. 2022, Misawa et al. 2022). This suppresses MtCEP1 expression which would otherwise positively regulate nodulation through MtCRA2 in the shoot. MtNRT2.1 is required for the peptide MtCEP1 to enhance nodulation and nitrate uptake (Fig. 3).

Most investigations on CEPs focusses on the uptake of NO_3_^−^ and in root and nodule development. However, work in *Arabidopsis* and rice have shown an involvement of MtCEPR1 and OsCEP6.1 in improving nitrogen use efficiency (NUE) and yield related traits (Sui et al. 2016; Taleski et al. 2020). In legumes, we know little about the involvement of CEP based NO_3_- signalling in managing yield and NUE. Understanding the role of *CLE-HAR1*, *CEP-CRA2* and their specific downstream signals for the coordination of root, nodule and shoot development in response to variable N availability of soil would provide new avenues for legume improvement.

### Post translation regulation

N transport activities are also affected by post-translational regulation involving phosphorylation. The phosphorylation at Threonine 101 plays a role in the dual-affinity activity of AtNPF6.3, NO_3_^−^ signalling and NO_3_^−^ dependent auxin transport (Ho et al. 2009; Bouguyon et al. 2015). This phosphorylation site is conserved in MtNPF6.8; however its regulative role in transport activity has yet to be revealed (Morère-Le Paven et al. 2011; Pellizzaro et al. 2014). The HATS activity of NRT2.1 is controlled by phosphorylation of different sites (Ser28, Ser501). N starvation of plants was observed when NRT2.1 is phosphorylated at Ser 28 but rapidly dephosphorylated upon NO_3_^−^ resupply (Engelsberger and Schulze 2012; Jacquot et al. 2020). The phosphorylation of NRT2.1 at Ser 28 by NURK1 kinase resulted in a low interaction of NRT2.1 with its partner protein NRT3.1. In contrast, phosphorylation at Ser 28 by an unknown kinase enhanced interaction with NRT 3.1 proteins, controlling NO_3_^−^ uptake activity (Li et al. 2020). Protein kinases, CIPK8 and CIPK23 both have been previously reported in NO_3_^−^ signalling as NO_3_^−^ inducible genes, downregulated in *Chl1* mutants (Ho et al. 2009). CIPK23 is known to phosphorylate the NO_3_^−^ transceptor NPF6.3 (CHL1/NRT1.1) thus negatively regulating the primary NO_3_^−^ response under low NO_3_^−^ concentrations, while in contrast CIPK8 kinase acts a positive regulator for the low-affinity NO_3_^−^ response (Ho et al. 2009) (Fig. 3f). All NRT2 transporters in *Arabidopsis* (except NRT2.7) have conserved Ser residues at or near the same position in all NRT members (Jacquot et al. 2020; Kotur et al. 2012) supporting the idea that post-translational regulatory mechanisms are of strategic importance for root NO_3_^−^ uptake. Unravelling the regulation of these processes in legumes will be important to better understand the activities of these transporters.

### Nitrate transporters managing N_2_ fixation and nodule *N* homeostasis

In most legumes, *N* for growth can come from the reduction of atmospheric N_2_ to NH_3_ via the bacterial enzyme nitrogenase located inside bacteroids within infected nodule cells (Ferguson et al. 2014). Ammonium is released from the bacteroid and transported across the symbiosome membrane into the cytoplasm where it is assimilated to glutamine and glutamate via the GS-GOGAT pathway. From glutamine, the path forward depends on the nodule type with amide amino acids generated in indeterminate nodules (pea, *Medicago*) and ureides exported from determinate nodules (soybean, *Lotus*).

For efficient N2 fixation, the translocation of *N* to different sections of the nodule is important for maintaining *N* homeostasis and for the delivery of N out of the nodule to support growth for the rest of the plant (Murray et al. 2016). The rate and quantity of NO_3_- transport into nodules can influence both nodulation (AON) and nodule development. One mechanism was proposed where NO_3_^−^ derived NO competitively influences the ability of leghemoglobin to bind oxygen and disrupt the delivery of oxygen to actively respiring bacteroids (Kanayama et al. 1990). The movement of NO_3_^−^ in nodules and to and from root cells is poorly understood. Several nitrate transporters have been shown to be induced in nodules, including members of the NPF and NRT2 families. Although high concentrations of NO_3_^−^ supresses nodulation, recent findings have shown that the maintenance of nodule NO_3_^−^ concentrations are important for nodule function. LjNRT2.4 is a plasma membrane localised HATS transporter suggested to be involved in NO_3_^−^ transport by root and nodule vascular tissues. Loss of function mutants reduce nodule NO_3_^−^ content, growth, N_2_-fixation activity and disrupt a putative NO_3_^−^–NO respiration cycle that involves infected cell mitochondria and bacteroids (Valkov et al. 2020). Under hypoxic conditions, these phenotypic changes were more obvious supporting the notion of the NO_3_^−^–NO pathway in mitochondria and bacteroids act as an alternative energy source for N2 fixation (Valkov et al. 2020). Another NPF upregulated in nodules is *LjNPF3.1* found expressed in cortical cells of both roots and nodules. Disrupting *Ljnpf3.1* results in a reduction in shoot growth, increased anthocyanin accumulation in stems and an impairment of N2-fixation activity (Vittozzi et al. 2021). Like LjNRT2.4, LjNPF3.1 may operate in the management of NO_3_^−^ transport to nodules. *LjNFP8.6* is upregulated in nodules and when impaired, results in a reduction in N2 fixation but no impact on nodule number or NO_3_^−^ dependent nodule inhibition (Valkov et al. 2017). The high-affinity NO_3_^−^ transporter (*MtNPF7.6*) is also expressed in vascular (pericycle, xylem and phloem) cells (Wang et al. 2020). Mutants (*Mtnpf7.6*) show defects in nodule vasculature development and a reduction in bacteroid nitrogenase activity, possibly through an accumulation of NO and a reduction in leghemoglobin expression. MtNPF7.6 is suggested to mediate NO_3_^−^ uptake from the soil to deliver low concentrations into developing nodules through a xylem to phloem vascular transfer. A similar mechanism was reported in rice which showed OsNPF2.2’s role in vasculature development of different organs in rice (Li et al. 2015). The high-affinity NO_3_^−^ transporter, MtNPF1.7 (MtLATD/NIP), has a role in nodulation where mutant plants (*Mtlatd*) produce defective nodules (Bright et al. 2005; Yendrek et al. 2010; Bagchi et al. 2012).

The range of NPF genes identified in legume nodules suggests their importance in managing the symbiotic partnership and highlights the intimate connection with cellular NO_3_^−^ through signalling, and the maintenance of NO_3_^−^ homeostasis. Further research is required to increase our understanding how NPF and NRT2 genes manage the complicated exchange of nutrients between root, nodule and rhizobia (Table 1).

**Table 1:** Candidate genes of nitrate transporters in legumes for NUE improvement

Gene	Plant	Function	Role in plant development	References
*MtNPF6.8*	*Medicago truncatula*	Dual affinity nitrate transporter	iLATS activity in plants, possible nitrate transceptor, primary root growth control	(Morère-Le Paven et al. 2011; Pellizzaro et al. 2014)
*MtNPF1.7*	*Medicago truncatula*	High affinity nitrate transporter	Root architecture and nodulation	(Bagchi et al. 2012)
*MtNPF6.5*	*Medicago truncatula*	Low affinity nitrate transporter	Chloride uptake/salinity stress	(Xiao et al. 2021)
*MtNPF6.7*	*Medicago truncatula*	Low affinity nitrate transporter	Nitrate uptake in roots	(Xiao et al. 2021)
*MtNPF7.6*	*Medicago truncatula*	High affinity nitrate transporter	Regulatory role in nodulation and function in nitrate uptake	(Wang et al. 2020)
*LjNPF2.9*	*Lotus japonicus*	Low affinity nitrate transporter	Downward transport of nitrate to root (nitrate distribution)	(Sol et al. 2019)
*LjNPF3.1*	*Lotus japonicus*	Low affinity nitrate transporter	Nitrogen fixation and nitrate uptake	(Vittozzi et al. 2021)
*LjNPF8.6*	*Lotus japonicus*	Low affinity nitrate transporter	Contributes in nodule functioning by controlling nitrogenase activity and nodular ROS content	(Valkov et al. 2017)
*MtNRT2.1*	*Medicago truncatula*	High affinity nitrate transporter	HATS activity in roots, regulation of nodulation	(Pellizzaro et al. 2015; Luo et al. 2023)
*MtNRT2.3*	*Medicago truncatula*	High affinity nitrate transporter	HATS activity in roots and nodule symbiosis	(Pellizzaro et al. 2015)
*LjNRT2.1*	*Lotus japonicus*	High affinity nitrate transporter	HATS activity in roots, regulation of nodulation	(Peng et al. 2019; Criscuolo et al. 2012; Misawa et al. 2022)
*GsNRT2.1*	*Glycine soja*	High affinity nitrate transport	HATS activity in roots	(You et al. 2020)
*LjNRT2.4*	*Lotus japonicus*	High affinity nitrate transporter	Role in nitrogen fixation in nodules and nitrate uptake	(Valkov et al. 2020)
*MtNRT3.1*	*Medicago truncatula*	Partner protein of NRT2	Nitrate uptake and mitigating arsenic contamination	(Ye et al. 2021) (Pellizzaro et al. 2015)
*LjNRT3.1*	*Lotus japonicus*	Partner protein of NRT2	Nitrate uptake in roots	(Peng et al. 2019) (Criscuolo et al. 2012)
*GmCLC1*	*Glycine max*	Chloride ion channel	Salt stress/homeostatic ionic balance	(Wei et al. 2016; Wong et al. 2013)
*GsCLC2*	*Glycine soja*	Chloride ion channel	Salt stress/homeostatic ionic balance	(Wei et al. 2019)

### Transport of nitrate in aerial tissues and its translocation to vegetative tissues

NO_3_^−^ transporters are increasingly being defined by their role in *N* redistribution between root and shoot tissues, and the transport of NO_3_^−^ between shoot and reproductive tissues (Wang et al. 2012). Their role can influence growth, enhance storage and tailor delivery at the cellular level. The first step for NO_3_^−^ delivery from root tissues to aerial plant parts is the unloading of NO_3_^−^ into the xylem. A few tissue specific gene expression studies in *Arabidopsis* have identified transporters that could play a role in NO_3_^−^ translocation within the plant (Iqbal et al. 2020; Tsay et al. 2007). The low-affinity nitrate transporter NRT1.5 (AtNPF7.3) was found expressed in pericyle cells near the xylem. Low rates of net NO_3_^−^ transport were observed in *nrt1.5* mutants with less NO_3_^−^ content found in the xylem sap. This suggests that this transporter exports NO_3_^−^ out of pericycle cells and loads into the xylem for its upward transport. Interestingly, these mutants showed normal NO_3_^−^ uptake when tissues were supplied much lower NO_3_^−^ concentrations. This implies yet another underlying mechanism is also involved in xylem loading to the shoot under variable *N* supply. So far only bidirectional NO_3_^−^ transporter activities where influx increases at pH 5.5 and efflux occurs at neutral pH have been observed. It would be interesting to know further about their role as efflux/export systems (Lin et al. 2008). In *Glycine max*, expression of *GmNRT1.5* was upregulated in roots under N starvation (You et al. 2020). The expression of another NPF member, AtNPF7.2 in xylem parenchyma cells highlights a role in xylem unloading. The increased root to shoot translocation of NO_3_^−^ in *nrt1.8* mutants makes this transporter a potential negative regulator of root to shoot NO_3_^−^ translocation (Li et al. 2010). A close homolog of this transporter, *GsNRT1.96* showed high expression in *N*-starved roots (You et al. 2020).

The basipetal transfer of NO_3_^−^ from shoot to root tissues has been shown to be influenced by the LATS NO_3_^−^ transporter *AtNPF2.9*, which is expressed in root phloem companion cells (Wang and Tsay 2011). Loss of *Atnpf2.9* activity disrupted the movement of NO_3_^−^ to the roots while enhancing root to shoot NO_3_^−^ transport (Wang and Tsay 2011). In *L. japonicus*, an ortholog of *AtNPF2.9*, *LjNPF2.9* was also found to be expressed in root vascular tissues including the pericycle and root phloem cells (Sol et al. 2019). In a knockout mutant (*Ljnpf2.9)*, shoot NO_3_^−^ content increased as did leaf area and shoot growth. The disruption of basipetal NO_3_^−^ transport did not impact the negative influence of NO_3_^−^ on legume nodulation but also didn’t disrupt N_2_-fixation capacities of the nodules (Sol et al. 2019). In *Glycine soja*, *GsNRT1.12* also shows a similar root expression pattern to *AtNPF2.9* (You et al. 2020). This commonality with *AtNPF2.9* expression and functional activities suggests GsNRT1.12 and LjNPF2.9 may also be important contributors to NO_3_^−^ homeostasis in legumes and that the redistribution of NO_3_^−^ from shoots to roots is important in regulating normal plant growth. It remains unclear what role NPF2.9 has on *N* delivery to support root growth (Fig. 2).

Once transported to the shoots, NO_3_^−^ is assimilated in the cytosol of leaf cells or stored in vacuoles depending on plant growth and stress conditions. A few transporters have been documented for this activity in *Arabidopsis*. *AtNRT1.4* (*AtNPF6.2*) is expressed in the leaf petiole. AtNRT1.4 is a low-affinity NO_3_^−^ transporter which when inactivated reduces the NO_3_^−^ content in the petiole while increasing in the leaf lamina. Petiole NO_3_^−^ content has been used to monitor *N* fertiliser demand in some plants (Keisling et al. 1995; Zhang et al. 1996). A major knowledge gap in legumes is our limited understanding of specific genes that are involved in N export from leaves.

AtNPF4.6 (AtNRT1.2) transports both NO_3_^−^ and ABA and is expressed in vascular tissues of leaves, hypoctyls, roots, imbibed seeds, and infloresence stems (Huang et al. 1999; Kanno et al. 2012). A recent study reported that AtNPF4.6 alters NO_3_^−^ partitioning within *Arabidopsis* leaves and following its accumuation in flower stalks of *atnpf4.6* mutants (Babst et al. 2019). Under low *N*, chlorophyll was increased in early developing mutant plants combined with a reduction in N export from mature leaves with an increase in the raceme. Due to the role of ABA in leaf senescence and *N* remobilisation, it is likely that *AtNPF4.6* regulates NO_3_^−^ transport from source leaves via ABA signalling. The high level of expression of NPF4 members (*GsNRT1.72* and *GsNRT1.43*) in *Glycine soja* leaves raises the possiblity that these transporters are involved in leaf NO_3_^−^ homeostatis. Further investigations are required to explore their role in the recycling of *N* metabolites in plants.

Limited *N* supply also motivates plants to transfer nutrients from older to younger leaves to support their growth. Both low and high-affinity transporters are involved in this remobilization. In this context, *AtNPF2.13* (NRT1.7) mobilises nitrate from old to young leaves via phloem loading (Fan et al. 2009). The expression of AtNPF2.13 was found in the phloem of minor veins of older leaves and the loss of NPF2.13 activity disrupted NO_3_^−^ transfer. Two other low-affinity NO_3_^−^ transporters found expressed in the companion cells of the major veins of leaves (*AtNPF1.1* and *AtNPF1.2*) also appear to influence the transfer of NO_3_^−^ from old to young leaves (Hsu and Tsay 2013). Transporters operating in the high-affinity ranges (*AtNRT2.4* and *AtNRT2.5*) may also participate in the phloem loading and translocation of NO_3_^−^ to aerial tissues. The N inducible, *AtNRT2.4* is expressed in the phloem of leaves and its loss of activity reduces leaf NO_3_^−^ content (Kiba et al. 2012). AtNRT2.5 is also thought to transport NO_3_^−^ to the shoots based on its expression pattern in the epidermis, cortex and minor veins of mature leaves (Lezhneva et al. 2014). In legumes, the expression of *MtNRT2.3*, *GsNRT2.1* and *GsNRT2.2* in shoots suggests a putative involvement in NO_3_^−^ transport from roots to shoots (Pellizzaro et al. 2015; You et al. 2020). The range of different NO_3_^−^ transporters involved in shoot NO_3_^−^ redistribution suggests a thorough investigation of similar genes in legume species is required to improve nitrogen use efficiencies when NO_3_^−^ is also available in the soil.

Recycling of *N* within plants is crucial to support the growth of new tissues and to ensure the development of reproductive tissues. In legumes, we have scarce information of the mechanisms and specific genes that regulate and facilitate N transport into and out of storage and reproductive tissues. This information will be required to help design strategies to improve NUE in legumes.

### Storage and remobilisation of NO_3_^−^ in reproductive tissues

Excess nitrate is often stored in vacuoles. Its subsequent remobilisation requires movement across the tonoplast into the cytosol and then transfer across the plasma membrane to be either assimilated during the transfer process or delivered via the phloem as NO_3_^−^ to developing tissues. AtCLCa/b transports NO_3_^−^ into vacuolar compartments. *AtClCa* is expressed in mesophyll cells of leaves and the removal of this gene led to reduced NO_3_^−^ content in the leaves (De Angeli et al. 2006; Geelen et al. 2000). Unlike *clca* mutants, no difference in nitrate contents have been observed for *clcb* mutants (von der Fecht-Bartenbach et al. 2010). Orthologues of these CLC members in legumes have been shown to regulate the NO_3_^–^/Cl^−^ ratio by mediating the uptake of Cl^−^ specifically (Wei et al. 2019, 2016).

In some plant species, NO_3_^−^ uptake is partially or completely inhibited during the reproductive stages of growth (Masclaux-Daubresse et al. 2010). In wheat, approximately ~ 90% of seed *N* comes from the remobilisation of stored canopy *N* (Kichey et al. 2007). A growing collection of NO_3_^–^ transporters have been identified from both the NPF and NRT2 families which mediate NO_3_^−^ redistribution and storage in seeds both at early and late developmental stages. *AtNRT2.7* is a gene encoding a high-affinity vacuolar NO_3_^−^ transporter that is highly expressed in seeds and in developing embryos and roots (Chopin et al. 2007). Mature seeds of *nrt2.7* mutants have less NO_3_^−^ content and freshly harvested seeds were more dormant than wild type and overexpressing plants. Depending on external supply, NO_3_^−^ also affects early seed development. The low-affinity NO_3_^−^ transporter, *AtNPF2.12* (*AtNRT1.6*) is expressed in vascular tissues of siliques and in the funiculus suggesting a role in delivering NO_3_^−^ to developing seeds. Early NO_3_^−^ delivery was found to be important at the one to four cell stage of early embryogenesis where loss of supply resulted in abnormal embryo development (Almagro et al. 2008). *Atnrt1.6* mutants showed increased abortion rates accompanied by less nitrate levels in the developing seeds (Almagro et al. 2008). The low-affinity NO_3_^−^ transporter (AtNPF5.5) has also reported to be involved in NO_3_^–^ transport into the embryo at the bent cotyledon stage of developing seeds (Léran et al. 2015). Similarly in legumes, You et al. (2020) showed that a NPF5 ortholog, *GsNRT1.71* was constitutively expressed in the pods of *Glycine soja,* suggesting a role in NO_3_^–^ transport or remobilisation in seeds. Legume seeds show high expression levels of the NO_3_^−^ transporters, *MtNPF4.12* and *GsNRT1.72*, during seed development and *LjNRT2.4* in mature seeds (Pellizzaro et al. 2017; You et al. 2020; Valkov et al. 2020). A recent study showed a role for *AtNPF7.1* in NO_3_^–^ transport to anthers and pollen based on its expression in flowers (Babst et al. 2019). Knocking out *AtNPF7.1* resulted in reduced rosette chlorophyll fluorescence and enhanced stalk growth compared to the WT controls. Accordingly, Babst et al. (2019) proposed a role for *AtNPF7.1* in N delivery to pollen grains or indirectly through *N* sensing in floral tissues.

### NO_3_^−^ transporters can mitigate multiple stresses in Legumes

Besides managing nitrogen availability, NO_3_^−^ transporters also help plants to cope with adverse environmental conditions (Wang et al. 2012). In legumes, CLC family members have been investigated for their ability in conferring salt tolerance. Overexpression of *GmCLC1* helped plants overcome salt stress, when seedlings were exposed to increasing concentrations of salt (50–150 mM). The transgenics grew better with significantly higher relative leaf water content and less relative electrolyte leakage than observed in WT plants. Moreover, the concentration of Cl^−^ ions in the roots of transgenic plants was lower than the controls (Wei et al. 2016). Using hairy root transformation, *GsCLc2* expressing plants appeared healthier with greater fresh weights, root vigour and relative water content than untransformed plants grown with 120 mM NaCl (Wei et al. 2019). A second study confirmed this result, showing *GsCLC2* was able to regulate root accumulation of both NO_3_^−^ and Cl^−^ (Liu et al. 2021).

As mentioned earlier, the transport of NO_3_^−^ occurs in combination with the transport of protons (H^+^), a process which can influence external pH (Miller et al. 2007). In acidic soils, higher H^+^ concentrations are toxic to some plants while overexpression of *AtNPF6.3* has been reported to confer tolerance to H^+^ toxicity via its NO_3_^−^ uptake activity (Fang et al. 2016). Cl^−^ in plants serves as an essential micronutrient required for regulating photosynthesis, stomatal movement, cellular turgor pressure and disease resistance. However, its excess limits absorption of important macronutrients, including *N*, *P* and *K* (Guo et al. 2014; Nguyen et al. 2016). The selectivity between NO_3_^−^ and Cl- by NPF proteins has recently been investigated. The *Zea mays* homolog of *AtNPF6.3*, *ZmNPF6.4* was shown to be selective to chloride uptake over NO_3_^−^ at low concentrations. This selectivity of *ZmNPF6.4* of Cl^−^ over NO_3_^−^ could be altered by introducing a His residue to replace a Tyr at AA 370 (His370Tyr) to make it NO_3_^−^ selective (Wen et al. 2017). A similar study in *M. truncatula* showed *MtNPF6.5* could transport Cl^−^ but be increasingly selective to NO_3_^−^ as external concentrations increased (Xiao et al. 2021). A second NPF (*MtNPF6.7*) was less prominent. This indicated Cl^−^ uptake activity for both MtNPF’s, with MtNPF6.5 like *ZmNPF6.4* as could behave as a Cl^−^ selective transporter. Under salt stress, *mtnpf6.5–3* mutants showed reduced Cl^−^ contents in roots and shoots (48–55% and 22–26%) than WT while *mtnpf6.7* mutants showed Cl^−^ levels to the WT. Longer primary roots with more lateral roots were developed in the *Mtnpf5.6* and *Mtnpf6.7* mutants under salt stress. However, in the presence of NO_3_^−^ these phenotypic changes were abolished. Recently, the expression of *GsNRT2.3, GsNRT2.4, GsNRT1.12, GsNRT1.43, GsNRT1.62* and *GsNRT1.57* were found to be upregulated when *Glycine soja* plants were treated with alkaline salts (NaHCO_3_) (You et al. 2020). These studies highlight the role of NPF’s in conferring salt tolerance in legumes as well.

The dual-affinity *AtNPF6.3* NO_3_^–^ also confers drought tolerance (Guo et al. 2003) consistent with its expression in guard cells of mature leaves and hypocotyls. Stomatal opening and transpiration rates were reduced in *Atnpf6.3* mutants under light/dark conditions making them more drought tolerant as compared to WT plants. Accordingly, Guo et al. (2003) proposed that a reduction in NO_3_^−^ uptake in guard cells of mutants during stomatal opening deteriorated guard cell depolarisation. Whether NPF6 homologs in legumes confer drought tolerance still needs to be investigated (Fig. 2). Waterlogging induces hypoxia in plants and elevates anaerobic respiration leading to a disruption in in the photosynthetic electron transport chain and the formation of reactive oxygen species (ROS). Recently (Valkov et al. 2020) have shown that during waterlogging, *LjNRT2.4* has maintained the normal functioning of nodules in *Lotus*. ABA has long been regarded as a stress hormone vital to plant biotic as well abiotic responses. The regulatory effects of exogenous ABA on high-affinity NO_3_^−^ transporters (HATS) have been observed in wheat roots (*TaNRT2.1*) (Taulemesse et al. 2015), while in *Glycine soja GsNRT2.1, GsNRT2.3, and GsNRT2.4* are upregulated when treated with different concentrations of ABA (You et al. 2020). In *Medicago*, *MtNPF6.8* has been shown to transport ABA in response to *N* limitation. Further investigations are needed to validate these findings.

Heavy metal contamination poses a great threat to human health due to the potential absorption and incorporation into the food chain (He et al. 2013; Zhao et al. 2010). *AtNPF6.3* confers cadmium tolerance while the loss of its activity results in a reduction in accumulated Cd under stress conditions in roots and shoots in the presence of NO_3_^−^ (Mao et al. 2014). A recent study showed that members of the NRT3 family in legumes, MtNRT3.1L1 (*MTR_4g104700*) and MtNRT3.1L2 (*MTR_4g104730*) help decrease arsenic (As) contamination in plants (Ye et al. 2021). The levels of accumulated arsenate [As (V)] were significantly less in *nrt3.1* mutants than WT. Absorption and accumulation of As (V) declined when the expression of *MtNRT3.1* was downregulated. Furthermore, complementation of *MtNRT3.1L1* in *nrt3.1* mutants showed that *NRT3.1* alone or via NRT2.1/NRT3.1 confers As (V) tolerance.

These studies suggest genes of nitrate transporters can become potentially new genetic targets for the future stress resistant legume crops using molecular breeding approaches.

## Conclusion

Studies across several model plant systems (*Arabidopsis, Medicago,* Soybean and *Lotus*) have greatly expanded our knowledge of the processes managing NO_3_^−^ transport to support both growth and seed development. The relationship between alternative *N* acquisition systems (N_2_-fixation and direct root uptake) are slowly starting to take shape identifying shared signalling pathways managing root and nodule development and the interdependency on alternative reduced N reserves to support early development of legumes subject to a rhizobial inoculation. The important next steps will be to define the regulatory controls limiting both nodulation and soil N to allow these systems to be used effectively together without penalties linked to carbon availabilities. Further work is required to understand the different layers of feedback mechanisms controlling N assimilation and the spatial (cell types) and temporal time periods they operate under. Genetic resources are improving in legumes, which will result in a rapid expansion of knowledge in this space to support the further growth and utilisation of legumes for sustainable protein production and the indirect benefits, through N deposition in the soil, weed and disease management afforded to other crops grown in rotation with legumes.

### *Author contribution statement*

ZR. and BNK conceptualised the idea, ZR, MNS and BNK performed the literature survey and wrote the draft manuscript, FP-W provided necessary suggestions, ZR, FP-W and BNK edited and critically evaluated the draft manuscript prior to submission.

## Data Availability

Data sharing is not applicable to this article as no datasets were generated or analysed during this current study.
